# Feasibility of hospital-initiated non-facilitator assisted advance care planning documentation for patients with palliative care needs

**DOI:** 10.1186/s12904-018-0331-3

**Published:** 2018-05-24

**Authors:** Maaike Kok, Gertruud F. M. van der Werff, Jenske I. Geerling, Jaap Ruivenkamp, Wies Groothoff, Annette W. G. van der Velden, Monique Thoma, Jaap Talsma, Louk G. P. Costongs, Reinold O. B. Gans, Pauline de Graeff, Anna K. L. Reyners

**Affiliations:** 1 Department of Medical Oncology, University of Groningen, University Medical Center Groningen, Hanzeplein 1, Groningen, 9700 RB The Netherlands; 2 Department of Internal Medicine, University of Groningen, University Medical Center Groningen, Hanzeplein 1, Groningen, 9700 RB The Netherlands; 3 Martini Hospital, Palliative Care Team, Van Swietenplein 1, Groningen, 9700 RM The Netherlands; 4 Ommelander Ziekenhuis Groningen, Palliative Care Team, Jachtlaan 50, Delfzijl, 9670 RA The Netherlands; 5 TSN Care Groningen, Dokter Stolteweg 60-66, Zwolle, 8002 LB The Netherlands; 6 Academic General Practice, University of Groningen, University Medical Center Groningen, Hanzeplein 1, Groningen, 9700 RB The Netherlands; 7 Zonnehuisgroep Noord, Izarstraat 1, Zuidhorn, 9800 AB The Netherlands; 8 University of Groningen, University Medical Center Groningen, Department of Internal Medicine, University Center of Geriatric Medicine, Groningen, 9700 RB The Netherlands

**Keywords:** Advance care planning [MeSH], Advance directive [MeSH], End-of-life care [MeSH], Palliative care [MeSH], Patient preference, Cohort studies, Retrospective studies

## Abstract

**Background:**

Advance Care Planning (ACP) and its documentation, accessible to healthcare professionals regardless of where patients are staying, can improve palliative care. ACP is usually performed by trained facilitators. However, ACP conversations would be more tailored to a patient’s specific situation if held by a patient’s clinical healthcare team. This study assesses the feasibility of ACP by a patient’s clinical healthcare team, and analyses the documented information including current and future problems within the palliative care domains.

**Methods:**

This multicentre study was conducted at the three Groningen Palliative Care Network hospitals in the Netherlands. Patients discharged from hospital with a terminal care indication received an ACP document from clinical staff (non-palliative care trained staff at hospitals I and II; specialist palliative care nurses at hospital III) after they had held ACP conversations. An anonymised copy of this ACP document was analysed. Documentation rates of patient and contact details were investigated, and documentation of current and future problems were analysed both quantitatively and qualitatively.

**Results:**

One hundred sixty ACP documents were received between April 2013 and December 2014, with numbers increasing for each consecutive 3-month time period. Advance directives were frequently documented (82%). Documentation rates of current problems in the social (24%), psychological (27%) and spiritual (16%) domains were low compared to physical problems (85%) at hospital I and II, but consistently high (> 85%) at hospital III. Of 545 documented anticipated problems, 92% were physical or care related in nature, 2% social, 5% psychological, and < 1% spiritual. Half of the anticipated non-physical problems originated from hospital III.

**Conclusions:**

Hospital-initiated ACP documentation by a patient’s clinical healthcare team is feasible: the number of documents received per time period increased throughout the study period, and overall, documentation rates were high. Nonetheless, symptom documentation predominantly regards physical symptoms. With the involvement of specialist palliative care nurses, psychological and spiritual problems are addressed more frequently. Whether palliative care education for non-palliative care experts will improve identification and documentation of non-physical problems remains to be investigated.

**Electronic supplementary material:**

The online version of this article (10.1186/s12904-018-0331-3) contains supplementary material, which is available to authorized users.

## Background

Currently, across Europe, almost half of palliative patients (49%) are hospitalised at least once in their last three months of life, with a readmission rate of 8% [1–3]. Many of these hospitalisations are thought to be avoidable [4–7]. In adjusted analyses, patients experience more physical and emotional distress at the end of life when hospitalised than at home, and they report lower QoL scores. Quality of death (as perceived by relatives) is also affected negatively [8–11].

Advance care planning (ACP) is “the ability to enable individuals to define goals and preferences for future medical treatment and care, to discuss these goals and preferences with family and health-care providers, and to record and review these preferences if appropriate” [12]. The use of ACP is correlated with a higher percentage of patients dying at the place of their choice, fewer hospital admissions and days spent in hospital in the last year of life and considerably lower healthcare costs [13]. In addition, they are associated with a better QoL and a reduced symptom burden [14–16]. This is achieved by timely, i.e. anticipatory end-of-life conversations and decision-making, followed by documentation [12–17]. Ideally, patients and their relatives are informed about the possible disease course, including future symptoms and situations within all four domains distinguished in palliative care, i.e. the physical/ care-related, social, psychological and spiritual domains. Patients can then make informed advance decisions consistent with their values, goals and preferences. Other important topics to be addressed are naming a proxy decision-maker and the use of advance directives with regard to hospital readmission, the use of aggressive and/or invasive treatments and cardiopulmonary resuscitation (CPR) [18]. ACP conversations should be documented in a dynamic document, accessible to all healthcare professionals involved with a patient at any time and place.

Because of the availability nowadays of a range of palliative treatments (e.g. surgery, chemotherapy, immunotherapy, advanced heart failure therapy), patients are treated at the hospital longer than they used to be, falling outside of the scope of the general practitioner (GP), traditionally the doctor to deliver palliative care [19–21]. Moreover, palliative patients are identified as such earlier on in their disease process. As a result, palliative care is initiated more frequently in the hospital setting, accompanied by the need for ACP conversations to be held there [20].

In a clinical setting, ACP conversations are frequently held by trained facilitators [19, 22–25]. Trained facilitators, sometimes referred to as care planning mediators, are persons with or without a clinical background who have been trained to engage in end-of-life conversations with patients, independently of the clinical team treating a patient [26–29]. However, to ensure patient tailored ACP conversations at the end-of-life as well as to ascertain the continuity of care throughout the palliative phase, hospital-initiated ACP conversations should ideally be held by a patient’s clinical healthcare team, in concertation with the GP.

To facilitate continuity of care throughout the hospital-to-home transition and improve patient handover for palliative patients, an ACP document, originally developed in a GP setting, was introduced in three hospitals [30]. The aim of the current study was to assess the feasibility of initiating ACP conversations and documentation at the hospital by the patient’s clinical healthcare team and analyse how ACP documents were filled out: whether information about patients, proxies and treating physicians had been documented and which current and anticipated future problems within each palliative care domain had been identified.

## Methods

### Patients and documentation

Within the province of Groningen, a rural region in the Northern Netherlands with a population of approximately 585,000, a regional network for palliative care was formed in 2013. The three hospitals (two district hospitals and a university hospital), community care organisations, nursing homes, hospices and GPs collaborate in providing palliative care. The ACP document (Additional file 1), based on Thoonsen et al. [30],was introduced in this network in April 2013 as part of a quality improvement project subsidised by the Netherlands Organisation for Health Research and Development. We conducted an observational study. Throughout the study period, whenever an ACP document was filled out at any of the three hospitals of this palliative care network, an anonymised copy was provided to be used for the current study.

Marking of the palliative phase and initiating ACP conversations was done by the patient’s treating physician. Reasons for initiating ACP conversations included deteriorating health, life expectancy < 3 months and/or fulfilment of the RADPAC criteria [30]. In two hospitals (I and II), including the university hospital, the ACP document was introduced at the medical oncology department. First, ACP conversations were held between patients, their relatives, treating physician, and the clinical nursing staff involved – in concertation with the GP. Following that, ACP documents were filled out by the patient’s clinical healthcare team. At hospital III, contrastingly, ACP documents were initiated and filled out by a specialist palliative care (SPC) nurse of the inpatient palliative care team (PCT) upon palliative care referral. ACP documentation in this latter hospital became structural from October 2014, following training of SPC nurses. ACP documentation only took place following verbal patient consent. The original ACP document stayed with the patient, to be consulted by all clinical and non-clinical healthcare staff caring for the patient, conditional on patient consent. According to Dutch Law, no ethical approval was required as participants were not subjected to any procedures on behalf of the research other than standard care.

### The ACP document

The ACP document consists of four separate single-page forms: (1) patient information including an overview of a patient’s advance directives; (2) an advance care plan stating anticipated future problems and an overview of all professional carers involved and how and when to contact them; (3) a current and recently discontinued medication overview and (4) a description of a patient’s current problems in all palliative domains: physical and care-related, social (including financial), psychological, and spiritual [21].

### Analysis

It was analysed how the hospital-initiated ACP documents were filled out by a patient’s clinical healthcare team (hospitals I and II) or an SPC nurse involved with a patient (hospital III). Items under study were (1) documentation of contact details: it was assessed whether a proxy, GP and treating hospital consultant and their telephone number had been documented. (2) Documentation of advance directives including cardiopulmonary resuscitation, hospital readmission, palliative sedation and euthanasia. For palliative sedation, it was documented whether patients had been informed about this medical intervention. Regarding euthanasia, it was documented whether a patient had brought up this subject. (3) An analysis of the documented current and anticipated problems in all palliative domains, both quantitatively and qualitatively. If mention was made on an ACP document that there were no current problems (rather than there being no documentation), this was noted down separately. The problem documentation rate was also analysed in time by comparing consecutive 6-month intervals.

Quantitative data were scored and analysed using IBM SPSS statistics 22. All current and anticipated problems, respectively, that were mentioned on all ACP documents, were recorded and scored individually. Identical problems signified with different terms, e.g. ‘pain’ and ‘ache’, ‘sickness’ and ‘nausea’, were grouped together by two independent researchers (MK and GvdW). Following that, problems were clustered into overarching problem categories for each palliative care domain by MK, with PdeG validating the categories independently. Prior to clustering, the anticipated problems were first classified into palliative care domains independently by GvdW and MK. In case of disparity, PdeG was consulted. Analysis of data was performed on the overarching problem categories that ensued.

## Results

### General descriptors

Between 1 April 2013 and 31 December 2014, 160 ACP documents were obtained. Most (*n* = 129; 81%) originated from hospitals I and II, and were filled out by non-palliative care trained staff (Table 1). The majority of patients (*n* = 136; 85%) had advanced cancer. Common cancers were lung (*n* = 23; 17%), pancreatic, hepatocellular/gall bladder and bowel (each *n* = 17; 13%). Non-oncological diagnoses occurred on a minority of ACP documents from all three hospitals (*n* = 24; 15%).

**Table 1:** Patient characteristics

Patient characteristic	*n* (%)
Male gender	81 (51%)
Mean age (years) ± SD	69 (±14)
Hospital of origin	
Hospital I & II^a^	129 (81%)
Hospital III^b^	31 (19%)
Diagnosis	
Cancer	136 (85%)
Chronic obstructive pulmonary disease	8 (5%)
Congestive heart failure	8 (5%)
Other (cholangitis (*n* = 1), peripheral arterial vascular disease (*n* = 2), a fractured femur (*n* = 1), septicemia (*n* = 1), end-stage renal disease (*n* = 2), stroke (*n* = 1))	8 (5%)

^a^ACP conversations and documentation carried out by non-palliative care trained staff treating/ nursing the patient

^b^ACP conversations and documentation carried out by an SPC nurse involved with the patient

Hospitals I & II

Hospital III

### Documentation of contact details

Contact details were filled out on more than half the ACP documents. A contact telephone number of a patient’s proxy was provided in 68% (*n* = 109). The proxy’s name (*n* = 87; 54%) and relationship with the patient (*n* = 94; 59%) were specified less often. GPs were named more often (*n* = 104; 65%) than their telephone number was provided (*n* = 92, 58%). Analogous to this, names of treating hospital consultants were documented more frequently (*n* = 141; 88%) than their telephone numbers (*n* = 110; 69%).

### End-of-life related issues

Advance decisions on hospital readmission (*n* = 130; 81%) and CPR decisions (*n* = 145; 91%) were frequently documented. At hospital III, documentation rates were 65% (*n* = 20) and 97% (*n* = 30), respectively. A majority of patients decided to forego further hospital treatment (*n* = 102; 64% overall; *n* = 17; 55% at hospital III). Most patients decided against CPR: *n* = 144 (90%) overall; *n* = 29 (94%) at hospital III. Conversations regarding palliative sedation and/or euthanasia were documented on 124 ACP documents (78%) overall; 28 documents (90%) at hospital III.

### Documented problems

The three most commonly documented problems or problem clusters for each domain are shown in Table 2, both for the current problems and the anticipated problems.

**Table 2:** Overview of current and anticipated problems

Domain	Physical	Care-related	Social	Psychological	Spiritual
*Current problems*					
Number of ACP documents with documentation (*N* (%))	136 (85%)	118 (74%)	39 (24%)	43 (27%)	25 (16%)
1	Pain (*n* = 58)	ADL dependence^a^ (*n* = 92)	Widowed/ living alone (*n* = 10)	Delirium (*n* = 19)	Acceptance (*n* = 7)
2	Dyspnoea (*n* = 42)	Urinary catheter (*n* = 11)	Worrying over loved ones (*n* = 9)	Mood problems other than anxiety (*n* = 17)	Problems with coping (*n* = 6)
3	Weakness/ fatigue (*n* = 33)	Faecal incontinence/ stoma (*n* = 7)	Impaired contact with family/ loved ones (*n* = 8)	Anxiety (*n* = 13)	Loss of control and independence (*n* = 4)
*Anticipated problems*					
Number of individual problems documented (N)	492	9	13	27	4
1	Pain (*n* = 114)	Physical weakness/ dependence (*n* = 6)	Overworked carer (*n* = 10)	Anxiety (*n* = 14)	Existential issues (*n* = 2)
2	Dyspnoea (*n* = 78)	Organisation-of-care related queries (*n* = 2)	Coping within family (*n* = 2)	Psychological restlessness (*n* = 4)	Coping (*n* = 1)
3	Nausea/ vomiting (*n* = 36)	Technical queries (e.g. Stoma care) (*n* = 1)	Unwanted loss of contact with child (*n* = 1)	Insomnia (*n* = 3)	Dilemma regarding pacemaker (*n* = 1)
Other	*n* = 264 Of which delirium: (*n* = 33)	Nil	Nil	*n* = 6	Nil

^a^Requiring help with activities of daily living, e.g. eating, bathing, dressing, toileting, transferring (walking) and continence

Physical and care-related current problem domains were filled out in a majority of cases: 88% (*n* = 141) and 78% (*n* = 125) ACP documents overall, respectively, and 97% (*n* = 30) and 94% (*n* = 29) at hospital III. This included documentation that there were no current problems in these domains: *n* = 5 (3%) for physical problems and *n* = 7 (4%) for care-related problems. For problems in the social and psychological/spiritual domains, documentation rates were substantially lower. On 83 ACP documents (52%), either social problems were documented (*n* = 39, 24%) or it was stated that there were no ongoing social problems (*n* = 44; 28%). ACP documents filled out by an SPC nurse resulted in more frequent documentation of social problems (*n* = 27; 87% for hospital III ACP documents). The psychological and spiritual domains were filled out on 101 ACP documents (63%) overall, with documentation rates of 57% (*n* = 73) from hospitals I and II and 90% (*n* = 28) from hospital III. Of these 101 ACP documents, 43 (of which 9 from hospital III) mentioned psychological problems, 25 (of which 5 from hospital III) stated problems of a spiritual nature, and on the remaining 33 ACP documents (of which 14 from hospital III) it was stated that there were no problems of a psychological or spiritual nature at the time of documentation.

A total of 545 anticipated problems were recorded, with a median of 3 (range 0–10) per ACP document. Of these anticipated problems, 501 (92%) were physical or care related in nature, 27 psychological, 13 social, 9 care-related and 4 spiritual. Almost half of these non-physical problems (25 out of 53) originated from ACP documents initiated in hospital III, including 9 out of 13 of the anticipated social problems.

### Analysis in time

When assessing the number of ACP documents received from hospitals I and II for each consecutive 6-month interval throughout the study period, a gradual and continued increase was noted (data not shown). Throughout the first 6-month interval, 21 ACP documents were received; the same 6-month interval one year later showed 46 ACP documents. Although no overall data regarding the number of patients eligible for ACP documentation were available, the subset of patient discharges from the medical oncology inpatient unit with a terminal care indication at hospital I as recorded by liaison nurses during both intervals remained stable at approximately 100–110 patients per year, implying an absolute increase in ACP documentation.

When looking at the documentation rate of current problems, there was an increase in documentation, particularly for the social and psychological/ spiritual domain, even though the total documentation rate remained low in hospitals I and II. The number of anticipated problems that was documented also showed a steady increase for each consecutive 6-month period, from a mean of 3.0 to 3.7 (data not shown).

## Discussion

### Main findings of the study

This study shows that an ACP document for palliative patients discharged from hospital, initiated by the clinical healthcare team treating the patient, is feasible. Upon its introduction, clinical nursing staff and medical doctors at the sites of introduction were instructed on the purpose and use of the ACP document. However, because of clinical rotations, the staff working with the document changed over time. Nonetheless, for each consecutive 6-month period from its introduction, the number of ACP documents that was filled out increased. Although no figures are known regarding the number of patients discharged with a terminal care indication at hospital II, this number remained stable at hospital I, showing the uptake of the ACP conversations and documentation in practice. Whilst initially not all eligible patients received an ACP document, throughout the study period, this portion kept increasing. Moreover, although the original indication for initiating ACP conversations and documentation was hospital discharge with a terminal care indication, throughout the study period, ACP documents were increasingly given to palliative patients with a longer life expectancy. This, too, demonstrates the uptake of ACP conversations and discussions in this setting.

The ACP document used was originally introduced in a study for GPs to timely identify palliative patients in their practice and initiate advance care planning [30]. Only half the participating GPs actually identified palliative patients as such, and these in turn represented only a fraction of the patients that had died expectedly during the study period. However, a post hoc analysis carried out by Thoonsen et al. of the GP palliative patient identification study they conducted, suggested that patients that had been identified had fewer hospital admissions, and died at home more frequently, suggesting a beneficial effect of ACP conversations and documentation. Because the majority of palliative patients are treated for their condition at a hospital, marking the palliative phase and concurrently initiating ACP conversations and documentation at the hospital, in concertation with the patient’s GP could lead to an increase in ACP. This, in turn, could lead to a better QoL for palliative patients towards the end of life. This is, however, conditional on the feasibility of hospital-initiated ACP by clinical healthcare staff.

Although several studies have shown the feasibility of ACP by trained and skilled facilitators, [22–25, 31] they are not as intricately aware of a patient’s specific situation and medical condition as the clinical healthcare team treating a patient. Many barriers to initiating ACP for clinicians have been identified [31, 32]. Contrastingly, this study shows that it is feasible for a patient’s clinical healthcare team to initiate ACP conversations and documentation. In this way, advance care planning becomes an integral part of patient care, and can be tailored to a patient’s specific situation and medical condition. This will allow for better coordination of palliative care because the ACP document functions as an additional, up-to-date, on-site handover document for all healthcare professionals involved with a patient.

From a qualitative perspective, hospital-initiated ACP documentation is feasible as well. Compared to a study assessing ACP documentation amongst internal medicine residents at two major UK academic teaching centres, twice as many health care proxies were established, and CPR decisions were also documented more frequently, in 91% versus 70% of cases [33]. On the other hand, fewer anticipated problems were documented in this study than patients are known to encounter towards the end of life [34, 35]. One study reported fewer problems towards the end-of-life for the hospital inpatient subgroup (mean 2.7 problems) than were anticipated in the current study. However, reported problems in that study were exclusively physical in nature, suggesting a possible bias due to under recognition of problems in the other domains [2].

In the current study, far fewer non-physical problems were documented than physical ones. This was particularly the case in hospitals I and II, where non-palliative care trained staff was responsible for ACP conversations and documentation. SPC nurses initiating ACP at hospital III seemed better equipped to identify non-physical problems. Likely, the importance and relevance of non-physical problems is not recognised by non-palliative care healthcare professionals, due to a lack of experience and training. Educating clinical healthcare providers could increase awareness and lead to a better recognition and treatment of these problems.

The majority of the ACP documents included in this cohort analysis were given to patients suffering from cancer. Of course, implementation took place at oncology departments in two of the three hospitals (hospitals I and II). However, it also reflects the fact that cancer is a common cause of death and has a relatively clear-cut course of decline and deterioration compared with for instance advanced chronic organ failure or dementia [36, 37]. In a survey among GPs, they report finding it more difficult to have end-of-life discussions with patients with organ failure and other non-cancer terminal diseases than with terminal cancer [19]. This may be true for hospital medical staff, too, but interestingly, despite the fact that ACP documentation was introduced exclusively at the medical oncology wards in hospitals I and II, advanced chronic organ failure was the diagnosis documented on 9 ACP documents originating from these hospitals, showing the uptake and spreading of ACP conversations and documentation in patients with non-cancer diagnoses.

The ACP document is not an end in itself. It is a dynamic document, onto which alterations can be made at any point by all clinical and non-clinical healthcare staff caring for a patient [38]. In this way, transcendence of the hospital-to-home boundary and vice versa is achieved. GPs, in close collaboration with community district nurses, can continue where the patient’s clinical healthcare team left off, ensuring continuity of care with the palliative patient at its centre.

### Strengths and weaknesses/ limitations

For this study, no information was gathered about patients’ or relatives’ views of the ACP document, nor did we obtain views of the GP and feedback about whether the document was used and if so, whether it was found helpful. It also remains to be researched whether this document will lead to fewer out-of-hours contacts, fewer unwanted hospital admissions and more deaths at the desired place of death.

Following the study period, the ACP document was evaluated by a working group of the regional palliative care network, based on how it was filled out by healthcare professionals. This led to some amendments to the document, for instance a change in wording: current and anticipated ‘problems’ were rephrased as ‘situation’, to be more inclusive. It was acknowledged that a complete picture of a situation comprises of more than problems alone.

### What this study adds

This study did demonstrate that initiating ACP conversations and documentation in a hospital setting is feasible, although there is room for improvement regarding the way in which the documents are filled out. An increased awareness of palliative care and advance care planning could be achieved by educating clinical healthcare staff. Education should focus on non-physical symptoms in particular; this may increase awareness and competence regarding the social, psychological and spiritual domains and improve holistic palliative care delivered to patients.

## Conclusions

This study shows that providing an ACP document to palliative patients discharged from hospital, initiated by the clinical healthcare team treating the patient, is feasible, both in terms of uptake and in terms of the quality of documentation. However, palliative issues regarding non-physical domains are underreported, except where palliative care trained staff are involved as part of a patient’s healthcare team. Education and training in ‘palliative reasoning’ might improve this knowledge gap.

## Additional file

Additional file 1: ACP_Document_Supplement_1. Handover document palliative care (English translation). This file contains an English translation of the ACP document that was used for this study. (DOC 93 kb)

## Abbreviations

ACP: Advance care planning
CPR: Cardiopulmonary resuscitation
GP: General practitioner
PCT: Palliative care team
SPC nurse: Specialist palliative care nurse
