# Supplementary material for: Feasibility of hospital-initiated non-facilitator assisted advance care planning documentation for patients with palliative care needs
Source: BMC Palliat Care. 2018 May 24;17:79. doi: 10.1186/s12904-018-0331-3 (PMC5967098; doi:10.1186/s12904-018-0331-3)
Supplement: Supplementary file 1 — ACP_Document_Supplement_1. Handover document palliative care (English translation). This file contains an English translation of the ACP document that was used for this study. (DOC 93 kb) [file 12904_2018_331_MOESM1_ESM.doc]

**Handover document palliative care**

**General information**

Patient name: Date of birth: male/ female

Address:

Post code: Town/ city:

Telephone number:

Social security number: Health insurance provider: Insurance number:

**Contact person (proxy):**

Telephone number:

**Treating consultant:**

Specialty:

Contact telephone number/ pager:

**Palliative team involvement yes/ no**

Contact person:

Telephone number:

**Legal guardian:**

Telephone number:

**Diagnosis:**

**Relevant medical history:**

**Advance directives/ decisions**

Hospital readmission (Yes/ No/ Conditional; please clarify)

Cardiopulmonary resuscitation and ICU admittance (Yes/ No/ Conditional; please clarify)

Euthanasia (discussed Yes/ No; if Yes: briefly state outcomes)

Palliative sedation (discussed Yes/ No)

**Comments**

**Advance Care Plan**

**Patient name:**  Date of birth**: male/ female**

| **Anticipated problems** | **Suggested solution** | **Evaluation** |
| --- | --- | --- |
|  |  |  |
|  |  |  |
|  |  |  |
|  |  |  |
|  |  |  |
|  |  |  |
|  |  |  |
|  |  |  |
|  |  |  |
|  |  |  |

| **Who to contact** | | |
| --- | --- | --- |
| **Role** | **Name** | **Telephone number** |
| General practitioner |  |  |
| Out-of-hours GP services |  |  |
| Consultant |  |  |
| Spiritual care professional |  |  |
| Psychologist |  |  |
| Other (specify which) |  |  |

**Medication**

**Patient name:**  Date of birth**: male/ female**

**Medication:**

**Allergies:**

| Date started | Discontinuation date | Drug and prescription | Rationale for discontinuation | Comments |
| --- | --- | --- | --- | --- |
|  |  |  |  |  |
|  |  |  |  |  |
|  |  |  |  |  |
|  |  |  |  |  |
|  |  |  |  |  |
|  |  |  |  |  |
|  |  |  |  |  |
|  |  |  |  |  |

**Comments**

**Current problems**

**Patient name:**  Date of birth**: male/ female**

| **Somatic domain** |
| --- |
| **Current problems:** |
| **Actions:** |

| **Social and financial domain** |
| --- |
| **Current problems:** |
| **Actions:** |

| **Spiritual and psychological domain** |
| --- |
| **Current problems:** |
| **Actions:** |

| **Care and ADL domain** |
| --- |
| **Current problems:** |
| **Actions:** |
